# Seed amplification and neurodegeneration marker trajectories in individuals at risk of prion disease

**DOI:** 10.1093/brain/awad101

**Published:** 2023-03-28

**Authors:** Tze How Mok, Akin Nihat, Nour Majbour, Danielle Sequeira, Leah Holm-Mercer, Thomas Coysh, Lee Darwent, Mark Batchelor, Bradley R Groveman, Christina D Orr, Andrew G Hughson, Amanda Heslegrave, Rhiannon Laban, Elena Veleva, Ross W Paterson, Ashvini Keshavan, Jonathan M Schott, Imogen J Swift, Carolin Heller, Jonathan D Rohrer, Alexander Gerhard, Christopher Butler, James B Rowe, Mario Masellis, Miles Chapman, Michael P Lunn, Jan Bieschke, Graham S Jackson, Henrik Zetterberg, Byron Caughey, Peter Rudge, John Collinge, Simon Mead

**Affiliations:** Medical Research Council Prion Unit at University College London, UCL Institute of Prion Diseases, London W1W 7FF, UK; NHS National Prion Clinic, National Hospital for Neurology and Neurosurgery, University College London Hospitals NHS Foundation Trust, Queen Square, London WC1N 3BG, UK; Medical Research Council Prion Unit at University College London, UCL Institute of Prion Diseases, London W1W 7FF, UK; NHS National Prion Clinic, National Hospital for Neurology and Neurosurgery, University College London Hospitals NHS Foundation Trust, Queen Square, London WC1N 3BG, UK; Medical Research Council Prion Unit at University College London, UCL Institute of Prion Diseases, London W1W 7FF, UK; Medical Research Council Prion Unit at University College London, UCL Institute of Prion Diseases, London W1W 7FF, UK; NHS National Prion Clinic, National Hospital for Neurology and Neurosurgery, University College London Hospitals NHS Foundation Trust, Queen Square, London WC1N 3BG, UK; Medical Research Council Prion Unit at University College London, UCL Institute of Prion Diseases, London W1W 7FF, UK; NHS National Prion Clinic, National Hospital for Neurology and Neurosurgery, University College London Hospitals NHS Foundation Trust, Queen Square, London WC1N 3BG, UK; Medical Research Council Prion Unit at University College London, UCL Institute of Prion Diseases, London W1W 7FF, UK; NHS National Prion Clinic, National Hospital for Neurology and Neurosurgery, University College London Hospitals NHS Foundation Trust, Queen Square, London WC1N 3BG, UK; Medical Research Council Prion Unit at University College London, UCL Institute of Prion Diseases, London W1W 7FF, UK; Medical Research Council Prion Unit at University College London, UCL Institute of Prion Diseases, London W1W 7FF, UK; Laboratory of Persistent Viral Diseases, Rocky Mountain Laboratories, National Institute for Allergy and Infectious Diseases, National Institutes of Health, Hamilton, MT 59840, USA; Laboratory of Persistent Viral Diseases, Rocky Mountain Laboratories, National Institute for Allergy and Infectious Diseases, National Institutes of Health, Hamilton, MT 59840, USA; Laboratory of Persistent Viral Diseases, Rocky Mountain Laboratories, National Institute for Allergy and Infectious Diseases, National Institutes of Health, Hamilton, MT 59840, USA; Department of Neurodegenerative Disease, UCL Institute of Neurology, Queen Square, London WC1N 3BG, UK; United Kingdom Dementia Research Institute at University College London, London WC1E 6BT, UK; United Kingdom Dementia Research Institute at University College London, London WC1E 6BT, UK; United Kingdom Dementia Research Institute at University College London, London WC1E 6BT, UK; United Kingdom Dementia Research Institute at University College London, London WC1E 6BT, UK; Dementia Research Centre, Department of Neurodegenerative Disease, University College London Queen Square Institute of Neurology, London WC1N 3AR, UK; United Kingdom Dementia Research Institute at University College London, London WC1E 6BT, UK; Dementia Research Centre, Department of Neurodegenerative Disease, University College London Queen Square Institute of Neurology, London WC1N 3AR, UK; United Kingdom Dementia Research Institute at University College London, London WC1E 6BT, UK; Dementia Research Centre, Department of Neurodegenerative Disease, University College London Queen Square Institute of Neurology, London WC1N 3AR, UK; United Kingdom Dementia Research Institute at University College London, London WC1E 6BT, UK; Dementia Research Centre, Department of Neurodegenerative Disease, University College London Queen Square Institute of Neurology, London WC1N 3AR, UK; United Kingdom Dementia Research Institute at University College London, London WC1E 6BT, UK; Dementia Research Centre, Department of Neurodegenerative Disease, University College London Queen Square Institute of Neurology, London WC1N 3AR, UK; United Kingdom Dementia Research Institute at University College London, London WC1E 6BT, UK; Dementia Research Centre, Department of Neurodegenerative Disease, University College London Queen Square Institute of Neurology, London WC1N 3AR, UK; Division of Neuroscience and Experimental Psychology, Wolfson Molecular Imaging Centre, University of Manchester, Manchester M13 9PL, UK; Department of Geriatric Medicine, Center for Translational Neuro- and Behavioral Sciences, University Medicine Essen, 45147 Essen, Germany; Department of Nuclear Medicine, Center for Translational Neuro- and Behavioral Sciences, University Medicine Essen, 45147 Essen, Germany; Nuffield Department of Clinical Neurosciences, Medical Sciences Division, University of Oxford, Oxford OX3 9DU, UK; Department of Clinical Neurosciences and Cambridge University Hospitals NHS Trust and Medical Research Council Cognition and Brain Sciences Unit, University of Cambridge, Cambridge CB2 7EF, UK; Sunnybrook Health Sciences Centre, Sunnybrook Research Institute, University of Toronto, Toronto, ON M4N 3M5, Canada; Neuroimmunology and CSF Laboratory, University College London Hospitals NHS Trust National Hospital of Neurology and Neurosurgery, London WC1N 3BG, UK; Neuroimmunology and CSF Laboratory, University College London Hospitals NHS Trust National Hospital of Neurology and Neurosurgery, London WC1N 3BG, UK; Medical Research Council Prion Unit at University College London, UCL Institute of Prion Diseases, London W1W 7FF, UK; Medical Research Council Prion Unit at University College London, UCL Institute of Prion Diseases, London W1W 7FF, UK; Department of Neurodegenerative Disease, UCL Institute of Neurology, Queen Square, London WC1N 3BG, UK; United Kingdom Dementia Research Institute at University College London, London WC1E 6BT, UK; Department of Psychiatry and Neurochemistry, Sahlgrenska Academy at the University of Gothenburg, S-43180 Mölndal, Sweden; Department of Psychiatry and Neurochemistry, Institute of Neuroscience and Physiology, The Sahlgrenska Academy at the University of Gothenburg, S-431 80 Mölndal, Sweden; Hong Kong Center for Neurodegenerative Diseases, Hong Kong, China; Wisconsin Alzheimer’s Disease Research Center, University of Wisconsin School of Medicine and Public Health, University of Wisconsin-Madison, Madison, WI 53792-2420, USA; Laboratory of Persistent Viral Diseases, Rocky Mountain Laboratories, National Institute for Allergy and Infectious Diseases, National Institutes of Health, Hamilton, MT 59840, USA; Medical Research Council Prion Unit at University College London, UCL Institute of Prion Diseases, London W1W 7FF, UK; NHS National Prion Clinic, National Hospital for Neurology and Neurosurgery, University College London Hospitals NHS Foundation Trust, Queen Square, London WC1N 3BG, UK; Medical Research Council Prion Unit at University College London, UCL Institute of Prion Diseases, London W1W 7FF, UK; NHS National Prion Clinic, National Hospital for Neurology and Neurosurgery, University College London Hospitals NHS Foundation Trust, Queen Square, London WC1N 3BG, UK; Medical Research Council Prion Unit at University College London, UCL Institute of Prion Diseases, London W1W 7FF, UK; NHS National Prion Clinic, National Hospital for Neurology and Neurosurgery, University College London Hospitals NHS Foundation Trust, Queen Square, London WC1N 3BG, UK

**Keywords:** prion, inherited, RT-QuIC, NfL, GFAP

## Abstract

Human prion diseases are remarkable for long incubation times followed typically by rapid clinical decline. Seed amplification assays and neurodegeneration biofluid biomarkers are remarkably useful in the clinical phase, but their potential to predict clinical onset in healthy people remains unclear. This is relevant not only to the design of preventive strategies in those at-risk of prion diseases, but more broadly, because prion-like mechanisms are thought to underpin many neurodegenerative disorders. Here, we report the accrual of a longitudinal biofluid resource in patients, controls and healthy people at risk of prion diseases, to which ultrasensitive techniques such as real-time quaking-induced conversion (RT-QuIC) and single molecule array (Simoa) digital immunoassays were applied for preclinical biomarker discovery.

We studied 648 CSF and plasma samples, including 16 people who had samples taken when healthy but later developed inherited prion disease (IPD) (‘converters’; range from 9.9 prior to, and 7.4 years after onset). Symptomatic IPD CSF samples were screened by RT-QuIC assay variations, before testing the entire collection of at-risk samples using the most sensitive assay. Glial fibrillary acidic protein (GFAP), neurofilament light (NfL), tau and UCH-L1 levels were measured in plasma and CSF. Second generation (IQ-CSF) RT-QuIC proved 100% sensitive and specific for sporadic Creutzfeldt-Jakob disease (CJD), iatrogenic and familial CJD phenotypes, and subsequently detected seeding activity in four presymptomatic CSF samples from three E200K carriers; one converted in under 2 months while two remain asymptomatic after at least 3 years’ follow-up.

A bespoke HuPrP P102L RT-QuIC showed partial sensitivity for P102L disease. No compatible RT-QuIC assay was discovered for classical 6-OPRI, A117V and D178N, and these at-risk samples tested negative with bank vole RT-QuIC. Plasma GFAP and NfL, and CSF NfL levels emerged as proximity markers of neurodegeneration in the typically slow IPDs (e.g. P102L), with significant differences in mean values segregating healthy control from IPD carriers (within 2 years to onset) and symptomatic IPD cohorts; plasma GFAP appears to change before NfL, and before clinical conversion.

In conclusion, we show distinct biomarker trajectories in fast and slow IPDs. Specifically, we identify several years of presymptomatic seeding positivity in E200K, a new proximity marker (plasma GFAP) and sequential neurodegenerative marker evolution (plasma GFAP followed by NfL) in slow IPDs. We suggest a new preclinical staging system featuring clinical, seeding and neurodegeneration aspects, for validation with larger prion at-risk cohorts, and with potential application to other neurodegenerative proteopathies.

## Teaser

Mok *et al.* present longitudinal biofluid RT-QuIC and neurodegenerative biomarker analyses of individuals at risk of and symptomatic for prion disease. RT-QuIC can detect seeding activity up to almost 4 years before disease onset, while neurodegenerative marker trajectories differ between fast and slow inherited prion diseases.

**See Minikel and Vallabh (https://doi.org/10.1093/brain/awad143) for a scientific commentary on this article.**

## Introduction

Prion diseases are transmissible and inevitably fatal neurodegenerative conditions characterized by recruitment of host-encoded cellular prion protein (PrP) into disease-associated polymeric assemblies which propagate by elongation and fission.^1^ The observed range of clinical and pathological expressions in humans, however, is strikingly heterogenous despite the shared fundamental disease mechanism.^2^ Sporadic Creutzfeldt-Jakob disease (sCJD), the most prevalent form, accounts for roughly 85% of the incidence of human disease, typically presenting with the triad of rapidly progressive dementia, ataxia and/or myoclonus. Inherited prion disease (IPD) caused by autosomal dominant highly-penetrant mutations in the prion protein gene (*PRNP*) comprises 10–15% of the incidence but produces a wide spectrum of clinical syndromes including CJD, fatal familial insomnia (FFI), Gerstmann-Sträussler-Scheinker (GSS) disease, peripheral PrP systemic amyloidosis from truncation mutations, and long-duration dysexecutive-apraxic syndromes seen in octapeptide repeat insertions (OPRIs).^3,4^ Acquired prion disease has historically attracted considerable media, political and public health attention, despite being the rarest manifestation. Relevant exposures include bovine spongiform encephalopathy prions in the diet, and use of blood and blood products for variant CJD (vCJD)^5^; cadaver-sourced human growth hormone,^6^ neurosurgery, and lyophilized dura mater in iatrogenic CJD (iCJD),^7^ and at mortuary feasts in the Eastern Highlands Province of Papua New Guinea in kuru.^8^

One of the most remarkable aspects of prion biology is the apparent long incubation phase between prion infection/exposure and disease onset, lasting up to five decades in kuru and cadaver-sourced human growth hormone-related iCJD.^6,9^ Prions are transmissible to laboratory rodents by inoculation allowing for study of the sequence of prion infection, propagation and toxicity, which forms two mechanistically distinct phases.^2 ,10 ,11^ Specifically, following inoculation, infectious prion titres rise exponentially to reach a plateau, which continues for a considerable time until disease onset. Infectivity and toxicity are therefore uncoupled, with the length of the plateau being inversely proportional to PrP expression level. If this two-phase kinetics model is applicable to human disease, the clinically silent incubation phase marked by high prion titres hypothetically offers a window of opportunity for discovery of fluid biomarkers that predict proximity to onset e.g. potential dynamic changes in measures of seeding activity and/or neurodegenerative markers. Moreover, if borne out in humans, the two-phase kinetics model would provide a foundation for targeted prevention strategies in prion disease.^12,13^

Longitudinal studies of defined populations with high lifetime risk of prion disease undoubtedly afford the best opportunity to elucidate the sequence of biomarker evolution during the presymptomatic phase in humans. For context, cadaver-sourced human growth hormone (c-hGH) was administered to at least 1849 UK individuals between 1958 and 1985, 81 of whom have so far succumbed to iCJD,^6,14^ while those currently at risk of IPD were estimated at 1000 in the UK.^15^ Accrual of longitudinal biofluid sample resources from these studies not only allows repeated examinations for biomarker discovery, but also for ascertainment of rates of change as an even more sensitive predictor of disease onset.^16^ It is not feasible to adequately power clinical trials for candidate drugs in prion disease prevention for a simple clinical end point,^17^ but the characterization of presymptomatic biomarkers could inform different strategies, enrichment in and learning from trials.

The advent of ultrasensitive real-time quaking-induced conversion (RT-QuIC) assays capable of detecting PrP-amyloid seeding down to the attogram (10^−18^ g) range in the last decade offers the potential to detect presymptomatic CSF PrP-amyloid seeding activity in at-risk individuals.^18,19^ The assay exploits the ability of PrP-amyloid in tested samples to convert recombinant PrP (rPrP) monomers within a reaction mixture, and accelerates the process with cyclical bursts shaking and rest to amplify rPrP amyloid fibrils; alteration of thioflavin T (ThT) emission spectrum from amyloid binding within the reaction mixture is then detected by a microplate reader in relative fluorescence units (RFU) over a certain threshold. Indeed, Orru *et al*.^18^ used RT-QuIC to demonstrate high levels of seeding activity in brains and CSF of hamsters experimentally inoculated with 263 K prions in the clinically silent incubation period before disease onset, paralleling prion bioassays in the two-phase kinetics model^20^; interestingly, Vallabh *et al*.^21^ identified presymptomatic RT-QuIC seeding activity in an single elderly carrier of the E200K mutation. CSF RT-QuIC assays in human prion disease to date have been honed primarily to detect sCJD and IPD E200K seeds to high sensitivity (>90%) and specificity (∼100%), far outstripping of its utility in other IPD disease syndromes.^22–27^ Nevertheless, assay developments along the way have identified key factors [incubation temperature, sodium dodecyl sulphate (SDS), Hofmeister salts, etc.] and novel seed-substrate compatibilities [truncated hamster (Ha90) and bank vole (BV) rPrPs], which may pave the way for optimizing RT-QuIC for the more fastidious seed species in IPD.^28–31^

Neurodegenerative biomarkers in prion disease are essentially downstream products of either neuronal injury, astrogliosis and inflammation, or other secondary disease pathologies. While none of them are strongly discriminatory between neurodegenerative diseases, particularly with cross-sectional values, the tracking of biomarker dynamics over time may segregate mutation carriers approaching disease onset from ageing effects in normal controls. The introduction of digital immunoassay platforms revolutionized biomarker detection sensitivity, now down to single molecule resolution (e.g. Singe molecule array, Simoa), instead of relying solely on overall chemiluminescence intensity.^32^ Recently, our Unit demonstrated segregation of plasma tau and neurofilament-light (NfL) levels between IPD mutation carriers from symptomatic IPD individuals, and more importantly showed rising levels in the 2 years prior to symptom onset in small numbers of converting individuals examined, through use of Simoa assays.^33^ Further advances in Simoa technology now allows for multiplex arrays measuring up to four candidate biomarkers, limiting depletion of precious biofluid resources.

The National Prion Monitoring Cohort (NPMC) study in the UK was well placed to address this unmet need, having recruited at-risk individuals with contemporaneous acquisition of longitudinal clinical, neuroimaging, neuropsychometric and neurophysiological data, along with assembling an expansive blood and CSF biofluid archive since 2008. In this study, we marshalled the combined utility of disease-specific PrP-amyloid seed amplification assay (RT-QuIC) and ultrasensitive multiplexed Simoa digital immunoassay platform to characterize biomarker discovery and evolution in individuals at risk of prion disease.

## Materials and methods

### Ethical statement and study participants

Biofluid samples from all at-risk and symptomatic prion disease used in this study were drawn from individuals enrolled into the NPMC with written consent. Blood samples were routinely drawn at each assessment from 2008 onwards, while acquisition of CSF samples started in 2015 following an amendment to existing ethical approval for the NPMC through the Scotland A Research Ethics Committee (05/MRE00/63).

The NPMC enrolled eligible individuals from October 2008 onwards, encompassing those symptomatic of all forms of prion disease (sCJD, iCJD, vCJD and IPD), asymptomatic individuals at risk of IPD (IPD-AR), iCJD (iCJD-AR) and vCJD, and healthy controls. The IPD-AR population includes confirmed asymptomatic carriers of pathogenic *PRNP* mutations, and untested blood relatives of those affected by, or known to carry, pathogenic *PRNP* mutations. The iCJD-AR population in the NPMC is composed of recipients of cadaver-sourced human growth hormone up to 1985. The schedule of assessments, and hence biofluid sampling intervals, were administered according to the stratum in which a participant falls, determined by the projected rate of disease progression,^34^ and by clinical need. At each assessment, research blood samples were also taken with written informed consent from willing friends or non-blood relatives as controls.

For Simoa biomarker comparison, healthy control CSF samples were sourced from the spouses and non-blood relatives of patients with young-onset Alzheimer’s disease (AD), the British 1946 Birth Cohort (Insight-46), CONFLUID cohorts (healthy controls with no cognitive concerns and Mini-Mental State Examination scores > 27), and NPMC (single at-risk individual subsequently mutation-negative on predictive testing); healthy control plasma samples and data were sourced from NPMC internally (friends and non-blood relatives of patients) and from non-mutation carriers within the Genetic Frontotemporal Dementia Initiative cohort (GENFI). For CSF RT-QuIC analyses, control samples were sourced from Institute of Neuroscience and Physiology at University of Gothenburg (individuals with AD confirmed by CSF biomarkers and other non-AD neurodegenerative diseases), NHNN Neuroimmunology Laboratory (CSF referred for non-neurodegenerative indications) and from NPMC (single healthy at-risk individual, subsequently mutation-negative on predictive testing).

### Proximity to clinical onset/conversion in IPD-AR individuals

Age at onset in IPD is highly variable (standard deviation ∼10 years even within a family), therefore many people who carry IPD mutations are healthy beyond their parental or average age of onset for each mutation. Consequently, we developed a new method to estimate the age of onset for IPD-AR, whereby each individual has an estimated age of onset in the future. This method approximates a cumulative normal distribution of risk for each mutation based on literature data, and sets estimated age of onset in the future equal to the accrual of 50% of an individual’s outstanding cumulative risk. Further details and an example are provided in the Supplementary material.

### NPMC biofluid sample processing

#### Blood

Whole blood samples collected in EDTA or citrate tubes destined for fractionation into plasma were centrifuged at 2000*g* for 10 min at room temperature (22°C) on arrival at the laboratory. The supernatant (upper plasma phase) was then divided into aliquots of 0.5–2.0 ml in Nunc Cryovials, and then frozen at −80°C.

#### CSF

CSF samples were collected in two separate polypropylene tubes (Sarstedt 62.610.018), designated as CSF-R (for RT-QuIC) and CSF-N (for neurodegenerative markers). CSF-R was divided into aliquots of 0.5–1 ml in Nunc Cryovials after gentle mixing. CSF-N was centrifuged at 2200*g* for 10 min at room temperature, and supernatant separated into aliquots of 0.5–1.0 ml in Nunc Cryovials. Both were then stored in −80°C freezers.

### Recombinant prion protein expression and purification

Full-length human [Hu rPrP; amino acid (aa) residues 23–231; accession M13899] and bank vole rPrP (BV rPrP; aa residues 23–231; accession AF367624), and truncated hamster (Ha90 rPrP; aa residues 90–231; accession K02234) and truncated bank vole rPrP (BV90 rPrP; aa residues 90–231; accession AF367624) were purified according to previously established methods.^35,36^ The full-length human P102L rPrP (HuPrP P102L rPrP; aa residues 23–231; accession M13899) construct contained His-tags, and as such was purified using a different protocol with some minor modifications.^37^ Further details are available in the Supplementary material.

### CSF RT-QuIC analyses

The standard RT-QuIC reaction mix per well was composed of 10 mM buffer (sodium phosphate pH 7.4, or HEPES pH 7.4 or 8.0), 130–300 mM NaCl or NaI, 0.1 mg/ml rPrP (Hu, BV, Ha90, BV90 or HuPrP P102L), 10 μM (ThT), 1 mM EDTA, and 0.001 or 0.002% SDS. Reactions were prepared in 96-well optical clear-bottomed plates (Nalgene Nunc International 265301). In each well, 80 or 85 µl of reaction mix was seeded with 20 or 15 µl of CSF, respectively, bringing the final volume up to 100 µl per well.

Thereafter, the loaded plates were sealed (Thermo Scientific Nunc 232702) and incubated in BMG FLUOstar Omega Lite or POLARstar Omega microplate readers between 42°C and 55°C, at double orbital shake/rest cycles of 60 s/60 s at 700 rpm. ThT fluorescence readings (excitation 450 ± 10 nm, emission 480 ± 10 nm; bottom read) were recorded at intervals of 45 min. Each sample was tested in quadruplicate and classed as positive if the relative fluorescence units (RFU) in ≥2 of 4 wells exceed the 10% baseline-corrected threshold within the corresponding time cut-off points.^38^ Samples initially resulting in 1 in 4 positive wells were retested, and if 1 in 4 wells remained positive, were classed as ‘equivocal’. Time cut-offs were determined by incubation temperature i.e. 50 h for 42°C, 30 h for 50°C, and 24 h for 55°C.^28^

### End point quantitation of CSF seeding activity

CSF seeding doses were determined through end point quantitation of RT-QuIC PrP-amyloid seeding activity using the Spearman-Kärber method originally used in animal bioassay.^36,39^ Each sample was serially diluted by one-third using a single non-prion control CSF sample to reconstitute the total seeding volume per well to 20 µl. We define 50% seeding dose (SD_50_) as a unit of seeding activity or end point sample dilution that yields positive responses in 50% (e.g. 2 of 4) RT-QuIC reaction wells according to the criteria above. The SD_50_ can be estimated from the results of a dilution series using:


(1) $$\mathrm{LogS}D_{50}=x_{\;p=1}+1/2d-d\sum p$$

where *x*_*p*=1_ being the highest log_10_ dilution with 4/4 positive wells; d = log dilution factor; *p* = proportion positive at a given dose; *∑p* = the sum of values of *p* for *x*_*p*=1_ and all higher dilutions. Adjustments can then be made to report SD_50_ per unit of neat sample, e.g. undiluted CSF. When a neat CSF sample (20 µl) yielded only 3/4 positive wells, the Spearman-Kärber method was not used, and instead, this sample was calculated to contain 1.5 SD_50_ (per 20 µl CSF) because, by definition, one SD_50_ gives 2/4 positive wells.

### N4PB biomarker measurement

Plasma and CSF glial fibrillary acidic protein (GFAP), NfL, tau and ubiquitin C-terminal hydrolase L1 (UCH-L1) were measured by Simoa using the N4PB kit on a HD-X Analyser (Quanterix), following the manufacturer’s protocol.^32^ In brief, samples were thawed and centrifuged at 10 000*g* for 5 min at room temperature (21°C) to precipitate any debris; subsequently, the samples were transferred to designated wells on the plates, diluted at 1:4 for plasma and 1:40 (or 1:100) for CSF with sample diluent, and bound to paramagnetic beads coated with capture antibodies specific for human GFAP, NfL, tau and UCH-L1. Longitudinal samples from a single patient where available, were analysed on the same plate. The biomarker-bound beads were then incubated with the respective biotinylated detection antibodies, which in turn are conjugated to streptavidin-β-galactosidase complex, which serves as a fluorescent tag. Hydrolysis of the complex of a resorufin β-D-galactopyranoside substrate results in a fluorescent signal proportional to the concentration of the respective biomarkers present. Measurements from each sample were with biomarker concentrations extrapolated from a standard curve, fitted to a four-parameter logistic algorithm. Coefficients of variation (CVs) were determined using four internal quality control samples, and were <20% and <10% for intra-assay and inter-assay comparisons.

Additional previously measured plasma NfL and GFAP values from GENFI non-mutation carriers were also used to supplement healthy control data; five samples from this group were also analysed in our study to validate that the inter-assay CVs were < 15% for these two markers.

### Data and statistical analyses

Similar to previous experience, the N4PB biomarker values including healthy controls were positively skewed.^33^ Log_10_ transformation of GFAP, NfL, tau and UCH-L1 values reduced skewness across our sample cohorts, rendering them approximately normally distributed, allowing group-wise comparison of means using single factor ANOVA followed by pairwise *t*-tests. To address the known normal ageing effects on GFAP, NfL and Tau levels,^40–43^ biomarker values for healthy control CSF and IPD-AR CSF greater than 2 years to predicted onset were normalized to age 60 (apart from UCH-L1, which did not demonstrate an age effect). Single factor ANOVA followed by pairwise *t*-tests (assuming α = 0.05) were then applied to compare means of age-normalized values grouped by the respective cohorts—healthy controls, IPD at-risk individuals more than 2 years to predicted/actual clinical onset (IPD-AR > 2 years), IPD at-risk individuals less than 2 years to predicted/actual clinical onset (IPD-AR < 2 years), symptomatic IPD individuals (IPD), sCJD/vCJD/iCJD individuals (CJD), and iCJD at risk individuals (iCJD-AR). Individual biomarker slopes were modelled using mixed effects models, with random effects of slopes and intercepts.

Confidence intervals (95% CI) for sensitivity and specificity of RT-QuIC assays were determined using the exact Clopper-Pearson interval.

Statistical analyses were carried out using GraphPad Prism (version 9.2.0) and STATA v15.1.

### Data availability

All raw data, including Simoa values and RT-QuIC relative fluorescence units, are available on request. Corresponding clinical data may be requested, but a data transfer agreement is likely to be required, including restrictions that protect confidentiality and consent terms.

## Results

The range of *PRNP* mutations in our combined IPD and IPD-AR biofluid cohorts in this study included 5-OPRI, 6-OPRI, P102L, P105S, A117V, Y157X, D178N-129V, D178N-129M, Y163X and E200K. For CJD, this included samples from sCJD, iCJD (cadaver-sourced human growth hormone) and vCJD; the iCJD-AR cohort only included recipients of implicated batches of cadaver-sourced human growth hormone. IPDs are further broadly classified as ‘fast’ or ‘slow’ according to mutation and its associated median/mean survival (fast IPD < 12 months e.g. E200K, D178N, etc); slow IPD > 12 months e.g. OPRIs, P102L, A117V, Y163X, P157X, etc.). We defined clinical conversion as the emergence of characteristic neurological symptoms and signs along with functional decline measurable by MRC Prion Disease Rating Scale scores, supported by the presence of mutation-specific investigation abnormalities e.g. diffusion weighted imaging abnormalities in E200K, neurophysiological abnormalities in P102L, polysomnographic abnormalities in D178N-FFI, etc.

### RT-QuIC PrP-amyloid seeding assay CSF sample cohorts

**Figure 1:**
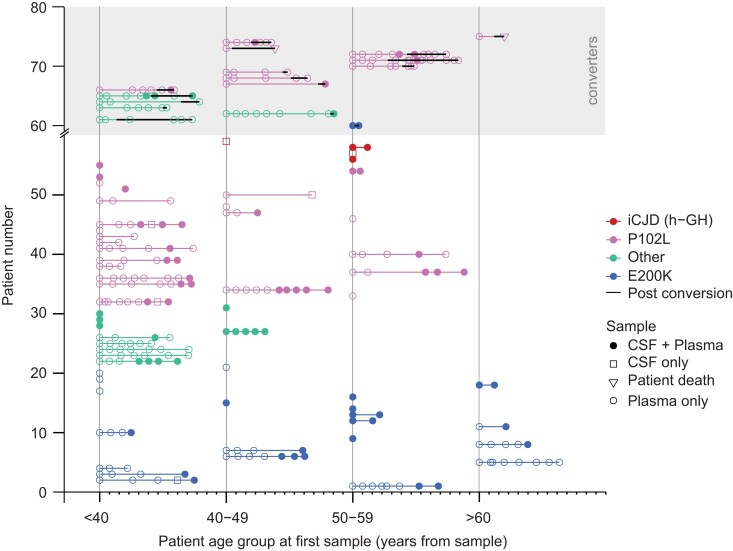
**IPD-AR, iCJD-AR and IPD converter biofluid sample archive.** This graph plots all the samples (plasma only, CSF only or matched plasma and CSF) analysed in this study, grouped by age category (<40, 40–49, 50–59 and >60) on the *x*-axis to obscure identities, with each minor tick mark after the start of each age category reflecting an interval of 1 year. A total of 12 years are covered per age group as the longest follow-up is over 11 years, in order to avoid overlapped timelines. The first (or only) sample from each individual is collapsed to the start of each age category to preserve anonymity. Samples from the same individual are joined by a horizontal line if more than one sample was collected; thick black horizontal lines denote onset of clinical conversion. Converters are grouped together in the upper shaded part of the graph. For converters where only one presymptomatic sample exists without any follow-up samples, the subsequent data-point (unfilled inverted triangle marker) joined by line indicates time of death. IPD mutations with fewer than five at-risk individuals were grouped as ‘Other’ to avoid self-identification.

From 2015 to 2021, 161 CSF samples were accrued for RT-QuIC analysis; IPD-AR samples account for the largest proportion (*n* = 61; individuals = 39), followed by IPD (*n* = 20; individuals = 20), sCJD/iCJD (*n* = 17; individuals = 17) and c-hGH iCJD-AR (*n* = 4; individuals = 3), which were tested against non-prion controls (*n* = 59; individuals = 59). Three pairs of samples exist from E200K, 6-OPRI and P102L converters, each with one sample before and after conversion. Baseline demographic details are summarized in Table 1. The entire at-risk and converter cohort is depicted graphically in Fig. 1.

**Table 1:** Baseline demographics of N4PB and RT-QuIC cohorts

					*PRNP* c129			
Cohorts/cohort subgroups	Number of samples	Unique individuals	Mean age at sample, y (SD)	F/M	MM	MV	VV	*PRNP* untested/unknown
**Plasma N4PB**								
IPD-AR	217	69	43.9 (13.3)	128/89	96	57	0	64
ȃIPD-AR >2 y	198	66	49.0 (13.2)	115/83	82	52	0	64
ȃIPD-AR <2 y	19	14	43.4 (13.3)	13/6	14	5	0	0
ȃP102L	100	33	43.7 (10.4)	68/32	26	25	0	49
ȃE200K	59	22	52.2 (15.9)	23/36	44	7	0	8
ȃMiscellaneous IPD	58	15	35.9 (9.17)	37.0/21	31	25	0	2
c-hGH iCJD-AR	3	2	55.7 (0.6)	1/2	1	0	0	1
Symptomatic IPD	62	26	50.9 (11.8)	40/22	33	29	0	0
CJD	40	18	52.0 (14.5)	12/28	16	19	5	0
Healthy controls								
ȃGFAP and NfL	132	84	49.7 (13.5)	66/66	0	0	0	127
ȃTau and UCH-L1	89	41	51.9 (13.1)	38/51	0	0	0	89
**CSF N4PB**								
IPD-AR	67	40	46.9 (12.4)	36/31	29	15	0	21
ȃIPD-AR >2 y	64	37	47.0 (12.2)	33/31	26	15	0	21
ȃIPD-AR <2 y	3	3	46.4 (19.2)	3/0	3	0	0	0
ȃP102L	30	16	46.0 (12.1)	18/12	6	5	0	19
ȃE200K	23	16	54.1 (10.7)	11/12	16	5	0	2
ȃMiscellaneous IPD	14	8	37.3 (8.4)	7/7	7	5	0	2
c-hGH iCJD-AR	5	4	53.8 (3.0)	1/4	1	0	0	4
Symptomatic IPD	22	21	48.4 (13.6)	14/8	14	8	0	0
CJD	17	17	60.6 (10.7)	10/7	7	9	0	1
Healthy controls	24	24	69.1 (6.8)	12/12	0	1	0	23
CSF RT-QuIC								
IPD-AR	61	39	46.5 (12.3)	31/30	28	13	0	20
ȃIPD-AR >2 y	58	36	46.5 (12.1)	28/30	25	13	0	20
ȃIPD-AR <2 y	3	3	46.4 (19.2)	3/0	3	0	0	0
ȃP102L	27	16	45.4 (11.7)	17/10	6	5	0	16
ȃE200K	22	16	53.5 (10.8)	10/12	15	5	0	2
ȃMiscellaneous IPD	12	7	36.2 (8.6)	5/7	7	3	0	2
c-hGH iCJD-AR	4	4	53.3 (3.1)	1/3	1	0	0	3
Symptomatic IPD	20	20	48.9 (13.4)	13/7	12	8	0	0
CJD	17	17	59.4 (10.5)	10/7	7	10	0	0
Non-prion controls	59	59	65.4 (14.5)	28/31	0	1	0	58

c129 = codon 129; F/M = female/male; MM = methionine homozygous; MV = methionine-valine heterozygous; SD = standard deviation; VV = valine homozygous.

### N4PB neurodegenerative marker sample cohorts

We assembled a total of 416 plasma and 135 CSF samples, from 2008 to 2021, for Simoa N4PB measurements. The IPD-AR cohort accounts for the majority of samples for both biofluids with 217 plasma samples from 69 unique individuals, and 67 CSF samples from 40 unique individuals. Crucially, this included longitudinal plasma (*n* = 86; individuals = 14) and CSF (*n* = 7; individuals = 3) samples capturing the interlude spanning clinical conversion (plasma range −9.9 to 7.4 years; CSF range −0.9 to 4.3 years); in two other IPD-AR individuals, a single plasma sample each was collected within 2 years of clinical conversion but none after. Of the 16 converted IPD individuals, eight had plasma NfL and tau levels measured by simplex Simoa platforms and published previously.^44^ In addition to the IPD-AR cohort, plasma (*n* = 3; individuals = 2) and CSF (*n* = 5; individuals = 4) samples from asymptomatic h-GH iCJD-AR individuals were also tested against symptomatic IPD, CJD (sCJD, iCJD and vCJD), and healthy control cohorts, with baseline demographics summarized in Table 1.

### Optimization RT-QuIC conditions for IPD CSF samples

A panel of CSF samples from clinically well characterized individuals with symptomatic prion disease [IPD and CJD (sCJD and iCJD)] were first screened with IQ-CSF RT-QuIC.^38^ Subsequently, an exploratory set of IQ-CSF RT-QuIC negative samples were put through iterative RT-QuIC assays with alterations in pH, buffer, incubation temperatures, salts, CSF seeding volumes, and rPrP species to determine the best available conditions for each IPD mutation, prior to testing the entire at-risk sample cohort.

Initial IQ-CSF RT-QuIC survey of the CJD sample set gave 15 positive (≥2/4 wells) and two equivocal (1/4 wells) results, with the equivocal samples becoming positive after adjustment of seeding volume from 20 µl to 15 µl. All four CSF from symptomatic E200K carriers were strongly positive with IQ-CSF RT-QuIC where all wells became positive within 10 h of incubation. CSF from a single 6-OPRI carrier drawn following an unexpected ‘CJD-like’ transformation with corresponding DWI changes on MRI brain indistinguishable from sCJD after several years of classical 6-OPRI disease progression, was also strongly positive (Supplementary Fig. 2). CSF from symptomatic P102L, P105S, D178N-129M, Y163X and classical 6-OPRI were all negative. All control CSF samples (*n* = 59) tested negative for IQ-CSF RT-QuIC with 20 µl seeding volume, as did all the control CSF (*n* = 47) using 15 µl seeding volume. Overall, the IQ-CSF RT-QuIC sensitivity and specificity for CJD and E200K IPD samples were both 100%.

A new bespoke variation of RT-QuIC using HuPrP P102L (PBS pH 7.4, 130 mM NaI, 0.002% SDS, 42°C) was positive in four of nine symptomatic P102L carriers. Of note, all four positive samples were from those with classical GSS phenotype at onset, though one underwent a ‘CJD-like’ transformation featuring typical DWI MRI brain changes after 2 years.^45^ CSF samples from three symptomatic P102L individuals with purely cognitive phenotypes and two with classical GSS phenotype tested negative with Hu P102L, wild-type Hu, BV, IQ-CSF RT-QuICs and all other exploratory conditions.

CSF seeding activity (3/4 wells) in a symptomatic P105S carrier with a CJD-like phenotype with cortical ribboning on DWI MRI Brain, was best demonstrated using Hu rPrP in pH 7.4 with 130 mM NaI at 42°C (Supplementary Fig. 3).

Optimum RT-QuIC conditions for D178N-129 M, Y163X and classical 6-OPRI were not found despite extensive exploration (Supplementary Table 1). In instances where seeding activity was demonstrated, they occurred beyond the cut-off time and frequently in close proximity to spontaneous fibrillization in control wells.

### RT-QuIC analyses of IPD-AR and iCJD-AR CSF cohorts

We divided the at-risk samples into the following groups, and matched them to the best available RT-QuIC assay determined in the exploratory phase: (i) E200K-AR and iCJD-AR to Ha90 rPrP in pH 7.4 and 300 mM NaCl at 55°C (IQ-CSF RT-QuIC); (ii) P102L-AR to HuPrP P102L in pH 7.4 and 130 mM NaI at 42°C (Hu P102L RT-QuIC) and BV rPrP in pH 7.4 and 300 mM NaCl at 50°C (BV RT-QuIC); and (iii) Other-AR to BV RT-QuIC.

**Figure 2:**
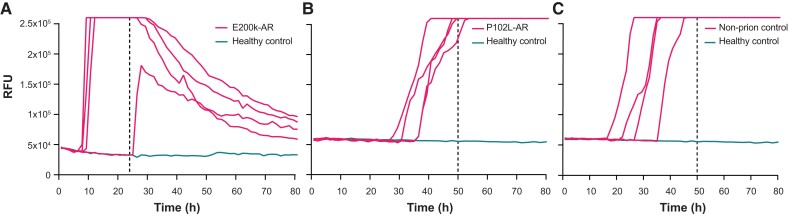
**Graphs of select IPD-AR and control samples with positive RT-QuIC results.** (**A**) This is the sole IQ-CSF RT-QuIC positive E200K-AR sample, which recorded fewer than 4/4 wells positive, drawn at 3.37 years from the present time. (**B**) This is the sole HuPrP P102L RT-QuIC positive sample in the P102L-AR set; this sample was negative when tested with BV RT-QuIC. (**C**) This non-prion disease (neurodegenerative) CSF sample tested positive with Hu P102L RT-QuIC, but tested negative with IQ-CSF RT-QuIC and BV RT-QuIC. The dotted vertical lines indicate the time cut-offs for the individual assays i.e. 24 h for IQ-CSF RT-QuIC and 50 h for Hu P102L RT-QuIC.

**Figure 3:**
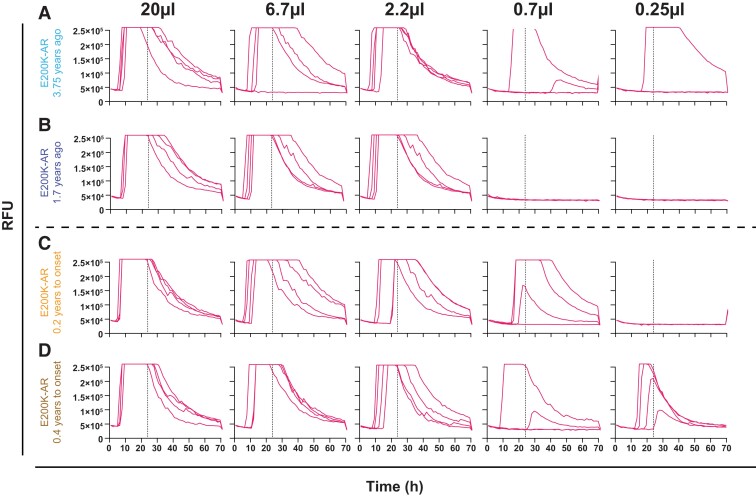
**RT-QuIC CSF end point dilutions for E200K-AR and E200K converter samples to calculate SD_50_/** **µl.** Each panel series show dilutions of seeding E200K CSF volume by a third; the vertical dotted line indicates the time cut-off, which was 24 h for IQ-CSF RT-QuIC. (**A** and **B**) From a single individual drawn at 3.75 and 1.70 years from the present, respectively. (**C** and **D**) From a single converter individual 0.6 years apart at 0.2 years to, and 0.4 years after conversion, respectively. The dotted vertical lines indicate the time cut-offs for the individual assays i.e. 24 h.

IQ-CSF RT-QuIC survey of the E200K-AR cohort (*n* = 22) revealed four positive results. All of these samples recorded 4/4 wells positive apart from one sample (remains asymptomatic at 3.37 years follow-up) in which 3/4 wells were positive (Fig. 2A). CSF SD_50_/µl estimates were calculated, as described in the ‘Materials and methods’ section. A pair from these samples belonged to an E200K converter, one 0.2 years before and the other 0.4 years after disease onset; the other pair was from an asymptomatic E200K carrier drawn at 3.75 and 1.70 years from the present time. The SD_50_/µl for the converter rose from 1.78 to 2.34, while that from the asymptomatic carrier dropped from 1.35 to 0.78 (Fig. 3).

In the P102L-AR subgroup tested with HuPrP P102L RT-QuIC, all samples were negative apart from one (21/22) sample from an asymptomatic at-risk untested individual over the age of 60; 1/57 non-prion control also tested positive (Fig. 2B and C). Both these samples remained positive on repeat testing; of note, this non-prion control sample tested negative in both IQ-CSF and BV RT-QuIC assays. The sole P102L-AR sample linked to a clinical converter, drawn 0.9 years prior to disease onset was negative, but the sample drawn 0.6 years after clinical onset tested positive (3/4 wells) with the HuPrP P102L RT-QuIC assay.

The Other-AR samples were tested with the BV RT-QuIC assay on the basis of BV rPrP being a potential ‘universal acceptor’ in experiments seeded by brain homogenates.^29^ None of the Other-AR samples (0/12) nor in the P102L-AR subgroup (0/27) tested positive with BV RT-QuIC. All the non-prion control CSF samples were negative (*n* = 51).

### Plasma Simoa N4PB results

**Figure 4:**
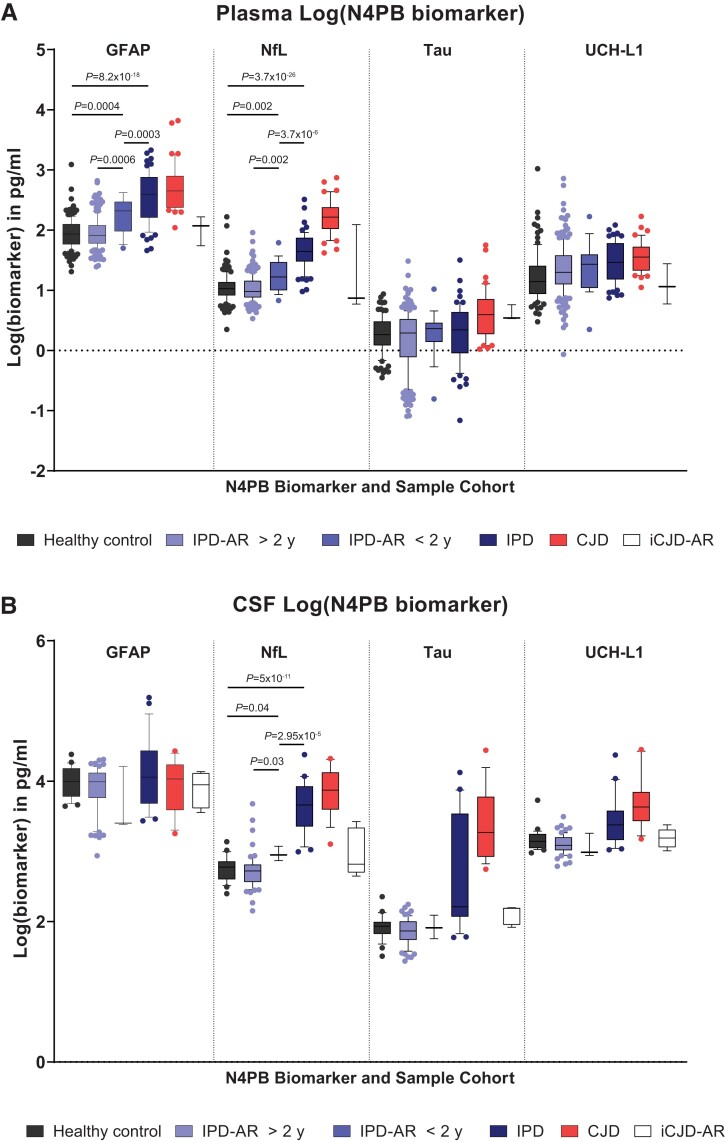
**Simoa N4PB measurements in prion disease and at-risk cohorts.** (**A**) Plasma N4PB levels, where only GFAP and NfL showed statistically significant different mean values between IPD at-risk and disease groups. (**B**) CSF N4PB levels, where only NfL showed statistically significant different mean values between IPD at-risk and disease groups.

Log(GFAP) and log(NfL) demonstrated sequentially incremental and statistically significant mean values between IPD-AR > 2 years and IPD-AR < 2 years, IPD and CJD cohorts on single-factor ANOVA with *post hoc* groupwise comparisons [Fig. 4A; log(GFAP), IPD-AR > 2 years versus IPD-AR < 2 years *P* = 0.0006, IPD-AR < 2 years versus healthy controls *P* = 0.0004, IPD-AR < 2 years versus IPD *P* = 0.0003; for log(NfL), IPD-AR > 2 years versus IPD-AR < 2 years *P* = 0.002, IPD-AR < 2 years versus healthy controls *P* = 0.002, and IPD-AR < 2 years versus IPD *P* = 3.7 × 10^−6^]. Of note, there were no significant differences in the mean values between the healthy control and IPD-AR > 2 years cohorts [*P* = 0.623 for log(GFAP); *P* = 0.298 for log(NfL)]. The mean values of the N4PB biomarkers according to cohort divisions, and the *P-*values from the single factor ANOVA analyses are summarized in Table 2.

**Table 2:** Mean values of age-normalized N4PB biomarkers according to cohort

Plasma N4PB	Sample number	Mean (pg/ml)	SD	ANOVA *P-*value	CSF N4PB	Sample number	Mean (pg/ml)	SD	ANOVA *P-*value
**Log(GFAP)**				1.72629 × 10^−60^	**Log(GFAP)**				0.109368
Normal control	132	1.94	0.25		Normal control	24	4.0	1.41	
IPD >2 y	198	1.95	0.26		IPD >2 y	64	3.9	1.40	
IPD <2 y	19	2.23	0.29		IPD <2 y	3	3.7	1.38	
IPD symptomatic	62	2.56	0.41		IPD symptomatic	22	4.1	1.42	
CJD	40	2.70	0.38		CJD	17	3.9	1.41	
iCJD-AR	3	2.01	0.25		iCJD-AR	5	3.9	1.40	
**Log(NfL)**				5.2614 × 10^−114^	**Log(NfL)**				5.91 × 10^−36^
Normal control	132	1.04	0.26		Normal control	24	2.8	1.29	
IPD >2 y	198	1.03	0.21		IPD >2 y	64	2.7	1.28	
IPD <2 y	19	1.26	0.27		IPD <2 y	3	3.0	1.31	
IPD symptomatic	62	1.65	0.29		IPD symptomatic	22	3.6	1.38	
CJD	40	2.22	0.29		CJD	17	3.8	1.40	
iCJD-AR	3	1.24	0.74		iCJD-AR	5	3.0	1.31	
**Log(Tau)**				6.99274 × 10^−6^	**Log(Tau)**				6.62 × 10^−28^
Normal control	94	0.26	0.30		Normal control	24	1.9	1.18	
IPD >2 y	198	0.18	0.49		IPD >2 y	64	1.9	1.17	
IPD <2 y	19	0.28	0.39		IPD <2 y	3	1.9	1.18	
IPD symptomatic	62	0.30	0.48		IPD symptomatic	22	2.6	1.27	
CJD	40	0.60	0.40		CJD	17	3.4	1.36	
iCJD-AR	3	0.61	0.13		iCJD-AR	5	2.1	1.20	
**Log(UCH-L1)^a^**				1.0087 × 10^−5^	**Log(UCH-L1)^a^**				5.84 × 10^−16^
Normal control	94	1.21	0.42		Normal control	24	3.2	1.33	
IPD >2 y	198	1.35	0.40		IPD >2 y	64	3.1	1.33	
IPD <2 y	19	1.38	0.42		IPD <2 y	3	3.1	1.32	
IPD symptomatic	62	1.48	0.33		IPD symptomatic	22	3.4	1.36	
CJD	40	1.55	0.26		CJD	17	3.7	1.39	
iCJD-AR	3	1.09	0.33		iCJD-AR	5	3.2	1.34	

Not age-normalized.

Mean age-normalized log(Tau) was not statistically significant between IPD-AR > 2 years versus IPD-AR < 2 years (*P* = 0.329), healthy control/IPD-AR > 2 years versus IPD (*P* = 0.1), and IPD-AR < 2 years versus IPD (*P* = 0.849). Mean log(UCH-L1) (not age-normalized) was not statistically different between IPD-AR > 2 years versus IPD-AR < 2 years (*P* = 0.802). As for the iCJD-AR cohorts, relevant statistically significant mean values were only seen with log(GFAP) against the CJD cohort (2.01 versus 2.70 pg/ml; *P* = 0.02), and with log(Tau) against normal controls (0.609 versus 0.264 pg/ml; *P* = 0.03); the latter was driven by a single outlier in the sample obtained from an iCJD-AR individual with contemporaneous destructive pituitary craniopharyngioma.

**Figure 5:**
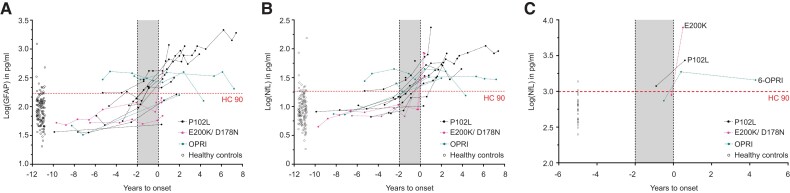
**Converter trajectories for plasma GFAP and NfL and CSF NfL.** Plasma GFAP (**A**) and NfL (**B**) trajectories are grouped into P102L (slow IPD) and E200K + D178N-FFI (fast IPDs) and OPRIs (slow IPDs; includes 5- and 6-OPRI). CSF converter data-points for NfL are shown in **C**. The horizontal dotted line indicates the 90th percentile of the respective biomarker value in the normal control cohort.

We identified 16 *PRNP* mutation carriers (P102L = 10, D178N-FFI = 2, E200K = 1, 5-OPRI = 1, 6-OPRI = 2) who underwent clinical conversion during follow-up over a median of 7.8 years [interquartile range (IQR) 5.2 years] in whom at least one presymptomatic plasma sample was available for analysis. An incline in plasma log(GFAP) and log(NfL) values was observed, but most consistently in P102L, D178N-FFI and E200K converting individuals. The pattern of log(GFAP) and log(NfL) evolution for the clinically fast IPD converters (E200K ± D178N-FFI)^33^ tend to exhibit relatively flat lines followed by abrupt rises close to or at the time of clinical onset. In comparison, the clinically slow *IPD* converters (P102L) showed a slower but more consistent upward trajectory in log(GFAP) and log(NfL) values, with 52.6% (10/18) and 44.4% (8/18) of measurements above the 90th percentile of healthy controls (HC90), respectively in the 2 years before clinical onset (Fig. 5A and B). The log(GFAP) and log(NfL) trajectories of the other slow IPD converters (one 5-OPRI and two 6-OPRI) were inconsistent, with two of three showing values above HC90 up to several years before the < 2 years window, and the remaining one 6-OPRI converter being below HC90 throughout. None of the iCJD-AR individuals had converted to iCJD on follow-up, but one died of invasive craniopharyngioma and another developed early-onset Alzheimer’s dementia.

We therefore modelled the linear trajectories of presymptomatic NfL and GFAP using mixed effects regression models with random effects for individual slopes and ‘fast IPD’ or ‘slow IPD’ as factor variables, including data prior to conversion (up to 4 years prior to conversion for slow IPD; 6 months for fast IPD) and one time point up to 6 months after conversion. These models estimated a slope for plasma log(NfL) of 0.108 pg/ml/year in slow IPD (95% CI 0.0662, 0.149) and 1.279 pg/ml/year in fast IPD (1.006, 1.551) with an *x*-intercept (time pre-conversion that linear modelled trajectory crosses mean of controls) of 2.448 years; for plasma log(GFAP) 0.090 pg/ml/year in slow IPD (95% CI 0.040, 0.140) with a *x*-intercept of 4.009 years and 0.458 pg/ml/year in fast IPD (95% CI 0.129, 0.787).

### CSF Simoa N4PB results

In the CSF cohort, only log(NfL) successfully demonstrated incremental, and statistically significant segregation of the mean values between the IPD > 2 years, IPD < 2 years and IPD stages (Fig. 4B; for IPD-AR > 2 years versus IPD-AR < 2 years *P* = 0.03, IPD-AR < 2 years versus healthy controls *P* = 0.04, and IPD-AR < 2 years versus IPD *P* = 2.95 × 10^−5^). No significant differences were shown between disease stages with log(GFAP). Statistically significant differences were seen for log(Tau) and log(UCH-L1) between IPD > 2 years and IPD < 2 years versus IPD, but not between IPD > 2 years versus IPD < 2 years.

In the three asymptomatic *PRNP* mutation carriers who clinically converted on follow-up, only one individual (P102L) registered a CSF log(NfL) value (3.07 pg/ml) above the HC90 (2.97 pg/ml) in the 2 years prior to clinical onset; all the other N4PB biomarkers in the remaining two converters were below the HC90 values (Fig. 5C). All three converters exhibited overall rises in N4PB biomarker levels after conversion, but not all exceeded the HC90 threshold. Correspondingly, in the matched plasma sample drawn at the same time as the CSF samples, all N4PB biomarker levels were below the HC90 for the E200K converter 0.2 years before clinical onset; for the 6-OPRI converter, both plasma log(GFAP) and log(NfL) were above the HC90 at 0.5 years prior to onset, while for the P102L converter, only plasma log(GFAP) exceeded the HC90 at 0.9 years prior to onset.

## Discussion

This study features extensive biomarker development in a large biofluid archive from individuals at risk of prion disease or in the early symptomatic stages. These data provide evidence for two types of fluid biomarker trajectory prior to clinical onset. First, not only did plasma GFAP emerge as a novel proximity biomarker, but linear increases initially in GFAP, and later NfL, in slow IPDs were detected up to 4 years pre-conversion. In contrast, in fast IPDs, the neurodegeneration biomarkers (NfL) change explosively around onset with no definable presymptomatic window. Second, in those IPDs for which we have highly sensitive seed amplification assays, particularly E200K, we found evidence of a presymptomatic CSF RT-QuIC seeding stage, considerably longer than the clinical phase of the disease (several years versus several months). These distinct aspects of prion pathophysiology are consistent with the two-phase kinetics model of prion propagation.^10^ They allowed us to envisage methods that might be used to stratify at-risk individuals and help the design and interpretation of presymptomatic treatment trials. It is important to note that *bona fide* prion infectivity that established the two-phase kinetics model may be distinct from PrP-amyloid seeding by RT-QuIC, in that RT-QuIC can be seeded by non-infectious aggregated PrP.

### E200K-AR biomarker trajectories

Our analytical approach was underpinned by the expectation that any RT-QuIC assay ought to be sufficiently sensitive for detecting seeding activity in symptomatic CSF samples in order to be able to do so in presymptomatic samples. The compatibility of IQ-CSF RT-QuIC for CJD and E200K seeds was confirmed in the symptomatic cohort, and then in the at-risk sample set by picking up four positive samples, one of which belonged to a subsequent converter. This argues that the previously reported asymptomatic positive RT-QuIC in an E200K carrier was not an isolated finding.^21^ In our cohort, no E200K at-risk individual converted without presymptomatic seeding activity. The E200K presymptomatic seeding period (as long as 3.75 years) appears unexpectedly long for an illness with such an explosive onset and short duration. The utility of tracking CSF SD_50_ values as a proximity marker remains unknown at present given the conflicting trajectories, and will require analyses of greater number E200K-AR follow-up samples and converters to fully elucidate. None of the presymptomatic RT-QuIC positive samples, including one drawn shortly before conversion, recorded abnormal neurodegeneration biomarkers, indicating that the onset of neurodegeneration is likely to be very close to conversion and potentially unrecognizable at current sampling intervals. As such, for those at incipient risk of clinical conversion, we expect asymptomatic positive CSF RT-QuIC (without evidence of neurodegeneration) to herald onset of biomarker evolution towards clinical conversion, though the intervals between seeding, neurodegeneration and clinical onset remain imprecise. Conversely, E200K carriers with negative CSF RT-QuIC and normal neurodegenerative markers may not be at risk of incipient conversion.

### P102L-AR biomarker trajectories

CSF from P102L affected individuals has historically been tested by variations of PQ-CSF and IQ-CSF RT-QuIC, usually included as very small subsets within large surveys of national CJD surveillance cohorts, with low sensitivities (Sano *et al.*^27^ is an exception; see the Supplementary material and Supplementary Table 1 for details).^23–26,46,47^ P102L individuals in these papers were classified as GSS with little information provided about clinical phenotype. Given the recognized phenotypic heterogeneity (classical GSS, cognitive and CJD-like) of P102L disease, and molecular evidence that these may be driven by distinct prion strains and possibly by non-infectious PrP amyloids accumulation, it is difficult to compare the results. It is quite possible that the few RT-QuIC positive samples reported may very well be due to the enrichment of individuals with the CJD-like clinical phenotype within surveillance cohorts.^45 ,48 ,49^

We developed a bespoke RT-QuIC assay using Hu P102L rPrP and NaI capable of detecting CSF seeding activity in a subset of P102L diseased individuals and a single untested at-risk individual over 60 years of age (3.85 years’ follow-up; all relevant N4PB values < HC90). Detailed phenotypic profiling suggest that this assay may work best in the P102L-GSS and P102L-CJD subgroups, but not in the P102L-Cognitive subgroup; due to the small numbers tested, it is unknown whether this observation will hold true when applied to larger P102L CSF sample sets. We speculate that the single RT-QuIC positive control sample may have belonged to an undiagnosed P102L patient, based on its highly selective amplification solely by the Hu P102L RT-QuIC (negative with IQ-CSF and BV RT-QuIC), and compelling kinetic curves, unlike the dubious false positives (‘slight amplification’ and ‘slowly rising curve’) reported in the literature.^50^ This sample was sourced from a clinical cohort with neurodegenerative symptoms referred for CSF examination, and subsequently classified as non-AD based on the biomarker profile; unfortunately no further details can be obtained as they were terminally de-identified. Despite its partial sensitivity, our assay may have a role in identifying a subset of at-risk individuals whose future conversion will be driven by compatible P102L PrP isoforms. As for neurodegenerative markers, plasma log(GFAP) and log(NfL) trajectories together with a considerable proportion [including CSF log(NfL)] being above HC90 in the 2 years before onset denote a longer pre-conversion phase of escalating toxicity relative to E200K. Slow IPDs may possess an extended pre-conversion seeding window if appropriately sensitive assays can be developed, but more clearly, show an identifiable presymptomatic neurodegeneration window.

### Other IPD-AR trajectories

The promise of BV rPrP as a ‘universal acceptor’ did not materialize during the RT-QuIC optimization phase, despite efforts to improve sensitivity. No presymptomatic CSF seeding activity was detected in classical 6-OPRI, P102L, A117V, D178N-129M and D178N-129V at-risk samples using BV RT-QuIC despite previous demonstrations that brain homogenates (10^−4^ dilutions) of all but D178N-129V cases can be detected using BV RT-QuIC.^29^ Neither plasma log(NfL) nor log(GFAP) appeared helpful in identifying D178N-129M individuals at risk of incipient conversion. Little conclusion can be drawn as yet from the inconsistent N4PB biomarker trajectories for our small number of 5-OPRI and 6-OPRI converters.

### Proposed presymptomatic IPD staging system

**Figure 6:**
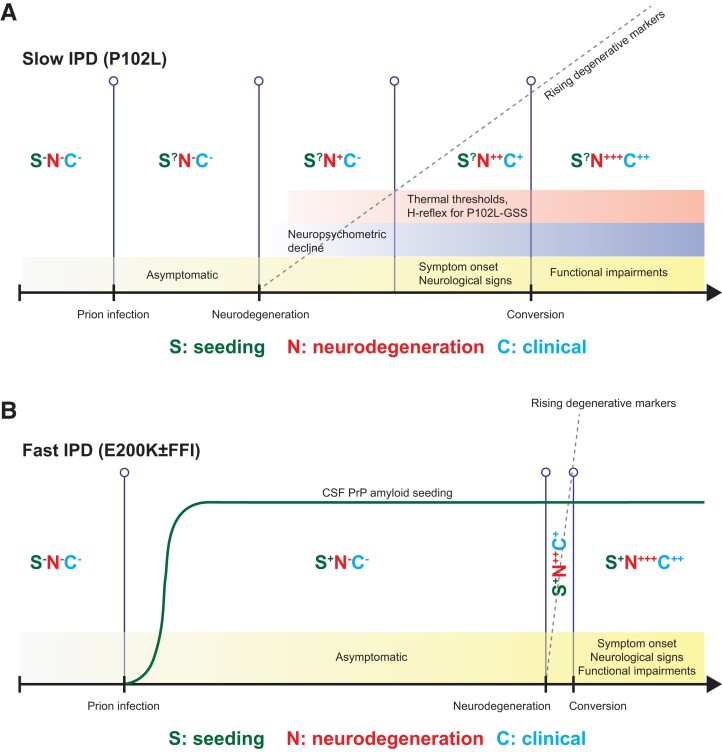
**Proposed pre-conversion IPD patterns of biomarker change for fast and slow IPDs.** Each stage features expected intensities in PrP-amyloid seeding activity, neurodegeneration markers and clinical aspects, along with ancillary investigations known to herald the onset of conversion (neuropsychometry, and neurophysiology in P102L). Naturally, the small numbers in this study precludes the provision of precise quantitative scales at the present time. (**A**) Slow IPDs are likely to have an extended window for neurodegenerative markers, making it easier to capture and follow at 6–12 monthly sampling intervals; however, we only have partially sensitive RT-QuIC seeding assays for slow IPDs. (**B**) Fast IPDs are likely to have a very short and explosive neurodegeneration window, which means it might not be easy to capture and follow at similar sampling intervals; this may be offset by the existence of highly sensitive RT-QuIC assays (E200K only) that may become positive several years before clinical onset. The changes in CSF PrP amyloid seeding are hypothetical, current evidence is limited to a very small number of individuals and samples.

We propose a general outline of presymptomatic biomarker change featuring key aspects of seeding activity, neurodegeneration, and clinical elements (Fig. 6). At this stage, despite new and consolidating evidence, we acknowledge this remains speculative, but provides a platform that can be tested and refined, as further data become available. Broadly, we saw patterns that vary for seeding and neurodegeneration between fast and slow IPDs. In fast IPD there is no useful presymptomatic neurodegeneration window at sampling intervals feasible in our study (Fig. 6). Neurodegeneration trajectories for slow IPD are easy to discern in retrospect, however we cannot yet be confident enough for individual prediction in isolation as values lie within the range of healthy controls. Accurate prediction for the purposes of individual feedback is self-evidently essential given that the information is so consequential. Counterintuitively, plasma biomarker dynamics appear to hold more promise than CSF, but this may be artefactual, merely reflecting the relative lack of sampling and follow-up data-points in the latter. The more immediate use may be for clinical trials, where we envisage the potential for biomarker-based enrichment of recruitment and biomarker outcomes in presymptomatic IPD. We believe that international collaboration will be essential to develop comparable sample collections with sufficient power to build confidence in these patterns of change.

### Relevance to other neurodegenerative diseases

If seeding assays can be more widely developed and applied in neurodegenerative diseases, as seems likely, these findings might provoke exploration of long presymptomatic seeding phases in other disorders. Discoveries in recent years have revealed fundamental aspects of common neurodegenerative diseases similar to prion diseases, particularly in proteopathic seed propagation, transmissibility and strain biology.^51–55^ The RT-QuIC-type proteopathic seed amplification assay borne out of the prion disease field has the potential to extend the presymptomatic stage earlier than the neurodegenerative phase, which is already very well characterized by imaging, neuropsychometric, fluid markers, etc. in AD and frontotemporal dementia.^16 ,56 ,57^ Indeed, the adaptation of RT-QuIC for α-synuclein has been used to probe the premotor phase of Parkinson’s disease, Lewy body dementia and multiple systems atrophy with success.^58–61^ Furthermore, RT-QuIC for 3-repeat, 4-repeat and AD tau, and even transactive response DNA binding protein-43 (TDP-43) are being honed for wider application in tissues and CSF.^62–65^

The extension of the presymptomatic phase to include a proteopathic seeding**-**only phase without evidence of neurodegeneration, will open even earlier windows of opportunity for intervention. This has particular implications on timing, and study design for therapeutic strategies against neurodegenerative diseases including human prion disease.

## Supplementary Material

awad101_Supplementary_Data
