# Supplementary material for: Seed amplification and neurodegeneration marker trajectories in individuals at risk of prion disease
Source: Brain. 2023 Mar 28;146(6):2570–83. doi: 10.1093/brain/awad101 (PMC10232278; doi:10.1093/brain/awad101)
Supplement: awad101_Supplementary_Data [file awad101_supplementary_data.pdf]

Supplementary Materials

1. Materials and Methods

a. Calculation of estimated time to disease onset

Assuming a normal distribution of age-related z scores against the probability density of clinical conversion, the *z* score associated with the current age (z_c) of the individual is first determined, from which the cumulative distribution function at z_c ($P(z < z_c)$) is derived. We then assume that the individual's residual cumulative risk (area under the curve) lies to the right of z_c which is the inverse i.e (1 – $P(z < z_c)$) and that the cumulative risk at z_p ($P(z < z_p)$) is the sum of $P(z < z_c)$ and half of its inverse (1 – $P(z < z_c)$)/2 i.e., $P(z < z_p)$ = $P(z < z_c)$ + (1 – ($P(z < z_c)$/2)). This can be expressed as a Microsoft Excel formula x_p =NORM.INV(1-(1-NORM.DIST(A2,A3,A4,TRUE))/2,A3,A4)-A2 where A2 is the individuals current age (x_c), A3 is the mutation mean age of onset, and A4 is the mutation standard deviation.

For example, the *z* score (z_c) of an E200K IPD-AR individual aged 60 (x_c) with a mutation mean (μ) of 58.5 and standard deviation (σ) of 8.0 years is calculated as follows:

$$z_c = \frac{x_c - \mu}{\sigma}$$

$$z_c = \frac{60 - 58.5}{8.0}$$

$$z_c = 0.1875$$

$P(z < z_c)$ of z_c (0.1875) is 0.5753, and therefore the inverse is 1 – 0.5753 = 0.4247. Half of 0.4247 is 0.4247/2 = 0.2123; and as such the z_p corresponds to $P(z < z_p)$ = 0.5753 + 0.2123 = 0.7876 is 0.8. Hence,

$$z_p = \frac{x_p - \mu}{\sigma}$$

$$x_p = (z_p \text{ x } \sigma) + \mu$$

$$x_p = (0.8 \text{ x } 8.0) + 58.5$$

$$x_p = 64.9$$

Finally, years to predicted onset is $x_c - x_p = 60 - 64.9$

$= -4.9$

Supplementary Figure 1 Determination of estimated age to onset for individuals at risk of prion disease (IPD and iCJD). (**A**) This shows the cumulative distribution function associated with the z score at the age of sampling ($P(z < z_c)$). (**B**) The total shaded area is the cumulative distribution function of the residual risk ($1 - P(z < z_c)$) and half of it is the estimated residual risk (**C**) for the individual at age of sampling ($1 - (P(z < z_c)/2)$). So (**D**) represents the cumulative distribution function of the age-related z score at estimated onset z_p i.e. $P(z < z_c) + (1 - (P(z < z_c)/2))$.

b. Recombinant PrP expression and Purification

For Ha90, Hu, BV and BV90 rPrPs, glycerol stocks of Escherichia Coli with vectors containing the respective *PRNP* sequences above (sourced from the NIH Rocky Mountain Laboratory) were used to inoculate cultures which were subsequently grown in Luria Broth medium together with kanamycin and chloramphenicol. The autoinduction system was then used to stimulate protein expression. Purification of rPrP from inclusion bodies in denaturing conditions was done through a Ni-nitrilotriacetic acid (NiNTA) superflow resin (Qiagen) with an ÄKTA Pure, before refolding through a guanidine HCl gradient and elution through an imidazole gradient. The eluted rPrP was sequentially dialysed extensively in 10 mM of sodium phosphate buffer pH 5.8, filtered through 0.22 µm syringe filter, its concentration determination by absorbance measurement at 280 nm, separated into aliquots, and frozen at -80°C. Prior to use, rPrP was thawed, filtered 100 kDa spin filter (Pall Nanosep), and concentration again similarly measured. The rPrP constructs purified with this method do not contain histidine tags (his-tags).

For Hu P102L rPrP, Escherichia Coli cultures containing the vector with this FL Hu P102L PrP sequence were grown Luria Broth medium, and in the presence of ampicillin. PrP expression was induced using Isopropyl β-D-1-thiogalactopyranoside (IPTG), and purified from inclusion bodies, similarly under denaturing conditions through NiNTA superflow resin (Qiagen) with an ÄKTA Pure, before refolding through a guanidine HCl gradient and elution through an imidazole gradient. The eluted rPrP was 1st dialysed extensively against 20 mM Bis Tris pH 6.5, and then had its his-tags cleaved by addition of 2.5 mM $CaCl_2$ and 50U Thrombin (VWR). The his-tags were when removed from the preparation by a 2nd run through NiNTA superflow resin (Qiagen) with an ÄKTA Pure, dialysed against 10 mM of sodium phosphate buffer pH 5.8, and treated exactly as above at its corresponding stage of handling.

2. RESULTS AND DISCUSSION

a. 6-OPRI case with positive IQ-CSF RT-QuIC

The abrupt change in clinical course of the 6-OPRI affected individual whose late-stage CSF tested strongly positive with IQ-CSF RT-QuIC, raises the possibility of a “strain switch”. The initial MRI Brain showed only atrophy, with CJD-like DWI changes emerging only after onset of rapid decline. We postulate that somehow, misfolded wild-type PrP became the dominant isoform, with the earlier phase of the illness being driven by classical 6-OPRI PrP isoforms. After all, 6-OPRI disease is known to present like sCJD from the outset and there are a handful of cases from the literature featuring less than a year’s disease duration (67, 68). These cases contained clinical synopses of variable detail, and were not accompanied by DWI MRI or RT-QuIC results, probably because they predate the advent of these technologies. Finally, co-propagation and contribution of misfolded wild type PrP has previously been demonstrated in P102L, where immunohistochemical discrimination between mutant and wild-type PrP is available.

Supplementary Figure 2 IQ-CSF RT-QuIC graph for 6-OPRI case with “CJD-like” transformation. This graph shows the positive RT-QuIC traces of individual wells against time for this individual with initial classical 6-OPRI phenotype followed by rapid deterioration and death. The dotted vertical line represents the time cut-off for this assay at 24 hours.

b. P105S case with positive Hu RT-QuIC with 130 mM NaI at 42°C

These RT-QuIC conditions appear to be able to detect seeding activity from our single symptomatic carrier of P105S who had a CJD-like clinical course of 3 months' duration; no presymptomatic CSF sample was available to test. The only other P105S case reported in the literature had a completely different clinical course with a disease duration of 10 years; initial MRI Brain showed only cortical atrophy (not clear if DWI was done) but another study 7 years later revealed typical CJD-like DWI changes. This other case predates the clinical application of the CSF RT-QuIC assay. Unlike E200K, it remains to been seen whether this tailored RT-QuIC assay using FL Hu rPrP with NaI is "universal" enough to capture and amplify all P105S related PrP isoforms to be used a diagnostic and/or presymptomatic screening assay.

Supplementary Figure 3 Hu RT-QuIC with 130 mM NaI versus 130 mM NaCl for P105S CSF. (**A**) This shows the RT-QuIC traces of individual wells seeded by CSF from an affected P105S individual, using NaI as salt in the reaction mix. This assay is more sensitive compared to using equivalent NaCl concentration as the salt shown in panel (**B**). The vertical dotted line indicates the time cut-off i.e. 50 hours.

c. Comparison of RT-QuIC assay conditions with Sano *et al.* 2013

Sano *et al.* 2013 reported a set of conditions for CSF RT-QuIC with high sensitivity and specificity for symptomatic E200K, P102L and D178N; the assay setup differs substantially from that used in this study (Rocky Mountain Laboratory (RML)). The key differences are summarised in Supplementary Table 1. We were unable to replicate the results from Sano *et al.* 2013, chiefly due to the different shaking kinetics between BMG and TECAN microplate readers.

Supp. Figure 1

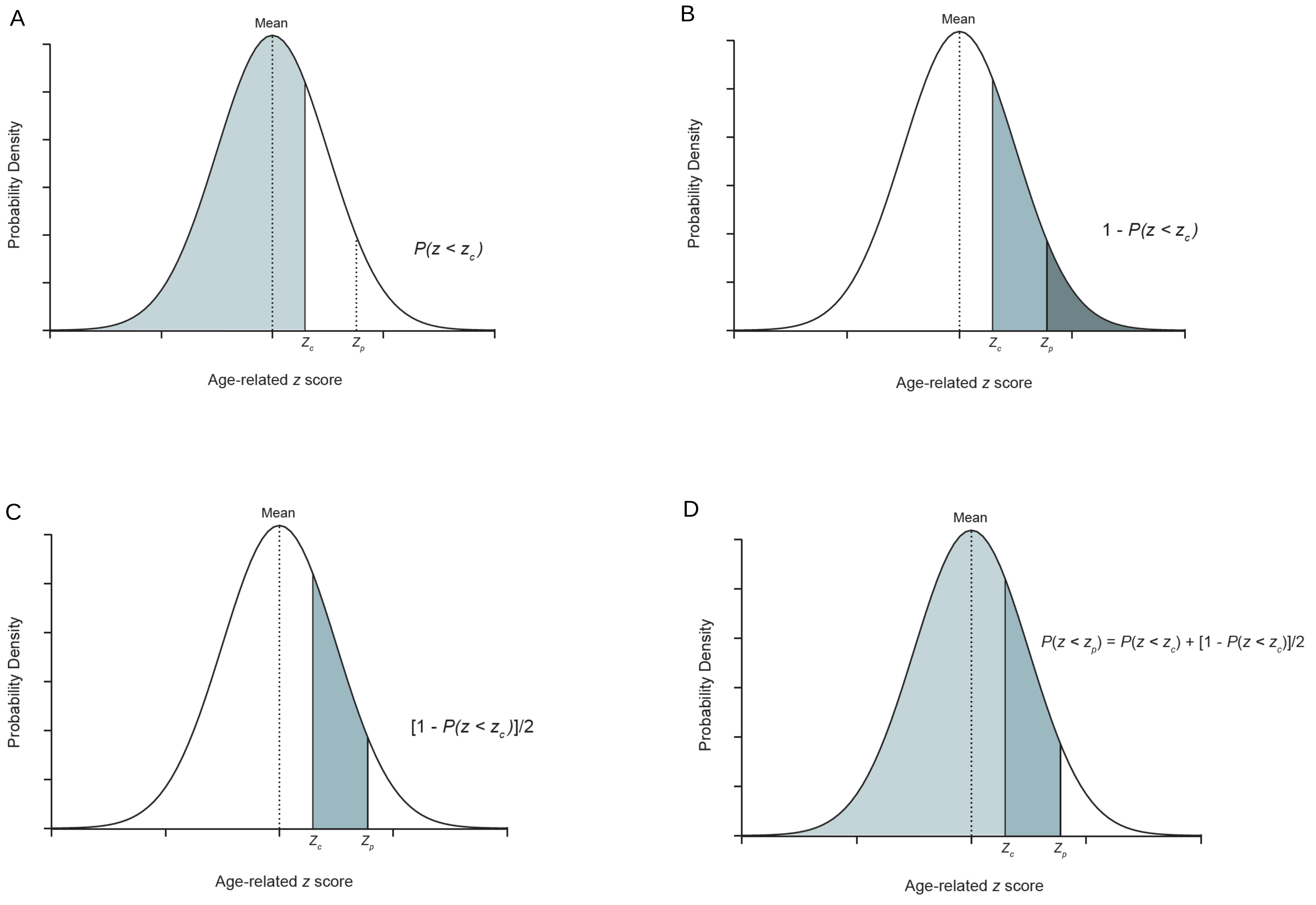

Supp. Figure 2

A

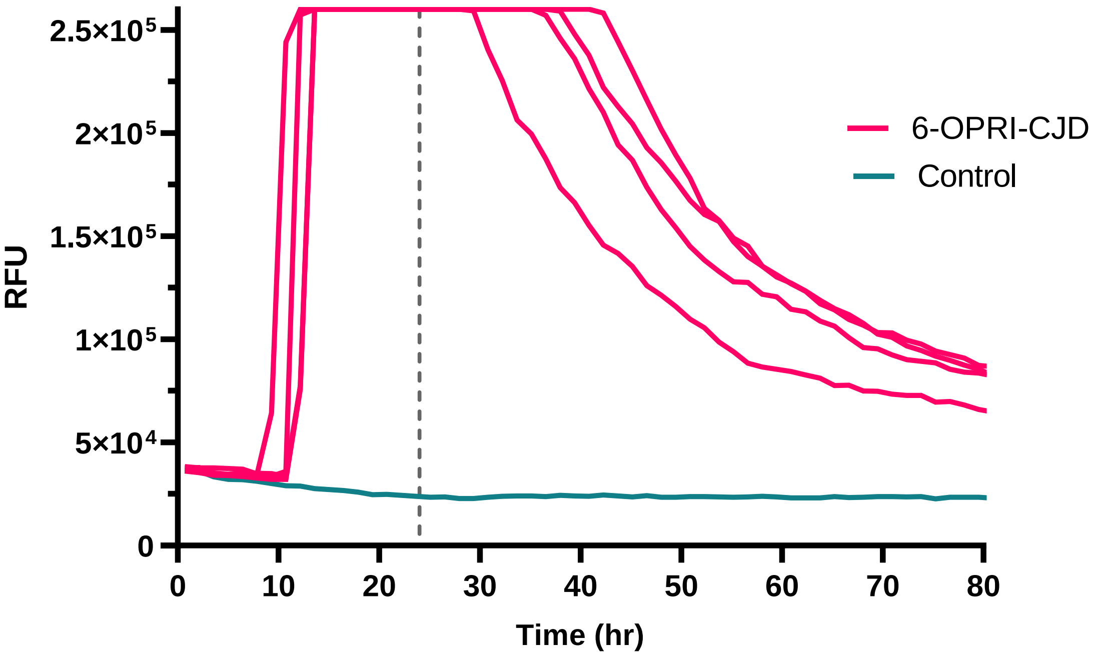

Supp. Figure 3

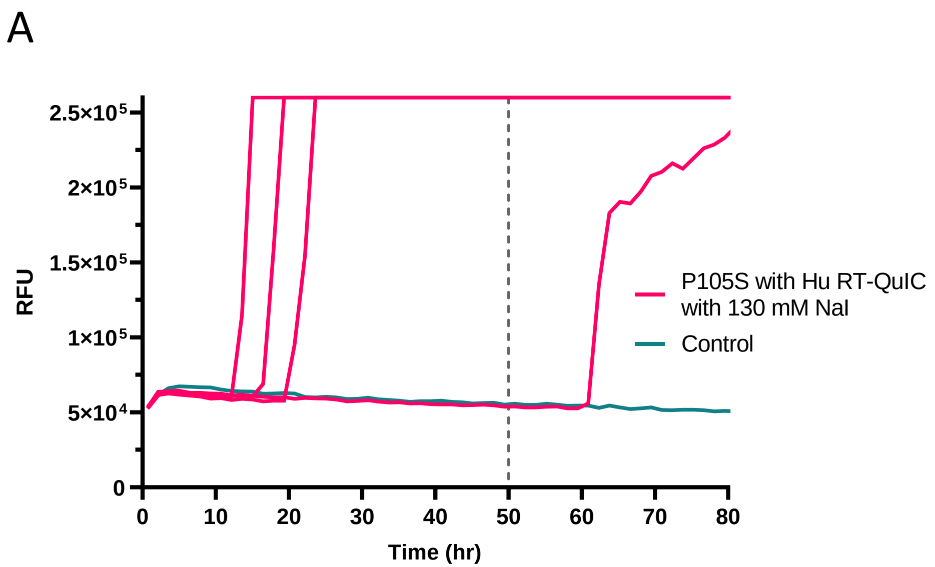

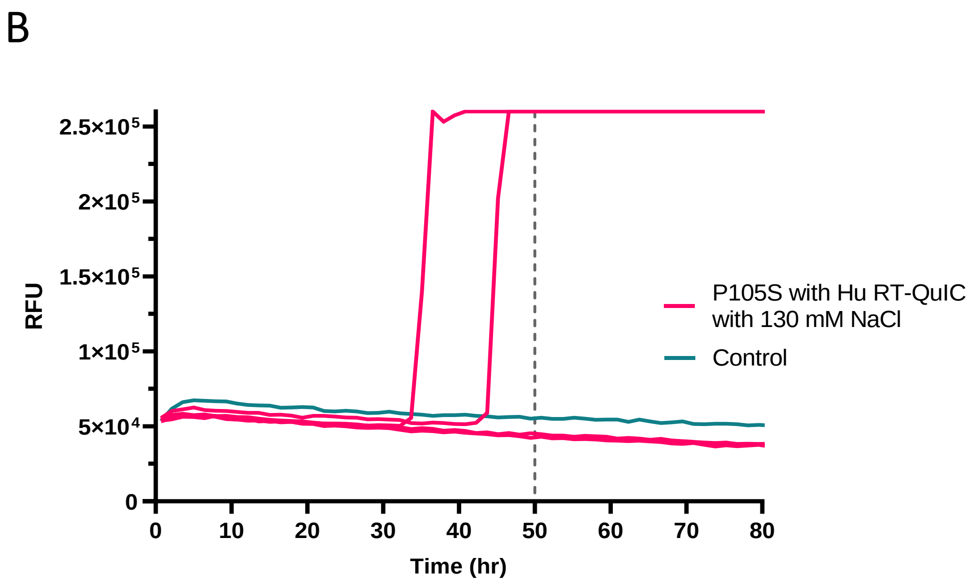

Supplementary Table 1 Comparison between RML and Sano et al. 2013 RT-QuIC assay setups

Conditions	RML	Sano *et al.* 2013
Buffer	10 mM phosphate buffer pH 7.4 (per well)	50 mM PIPES buffer pH 7.0 (per well)
Salt	NaCl 300 mM or NaI 130 mM (per well)	NaCl 500 mM (per well)
EDTA	1 mM (per well)	1 mM (per well)
SDS	0.002% (per well)	NA
ThT	10 mM (per well)	10 mM (per well)
rPrP species	Ha90, BV or Hu P102L	Wild type Hu
rPrP concentration	0.1 mg/ml (per well)	0.05 mg/ml (per well)
CSF seeding volume	20 or 15 µl (per well)	5 µl (per well)
Total volume	100 µl (per well)	100 µl (per well)
Microplate reader	BMG FLUOstar or POLARstar	TECAN M200 or F200
Shaking speed	700 rpm	Maximum speed (432 rpm; 1mm amplitude)
Shaking motion	Double orbital	Circular
Shake/rest intervals (on/off)	60s/60s	30s/30s + 2 min pause
Incubation temperature	55°C, 50 °C or 42 °C	37 °C
Time cut-off	24 hrs for 55°C; 30 hrs for 50 °C; 50 hrs for 42°C	53 °C (?)

Supplementary Table 2 Exploratory RT-QuIC conditions for symptomatic IPD CSF panel

rPrP	rPrP (mg/L)	Temp °C	Buffer	pH	Salt	Salt (mM)	SDS %	Cut-off (hrs)	CSF (µl)	Notes
Ha90	0.1	55	PBS	7.4	NaCl	300	0.002	24	20	No spontaneous fibrillisation in Control CSF wells up to 85 hrs; highly sensitive for E200K and CJD-like 6-OPRI
Ha90	0.1	55	PBS	7.4	NaI	300	0.002	24	20	Control CSF 2/4 wells positive ≥ 25 hrs; unstable
BV	0.1	55	PBS	7.4	NaI	300	0.002	24	20	Control CSF 2/4 wells positive > 55 hrs; negative for P102L, D178-FFI, 6-OPRI, Y163X
BV	0.1	50	PBS	7.4	NaCl	300	0.002	30	20	No spontaneous fibrillisation in Control CSF wells up to 85 hrs; negative for P102L, D178-FFI 6-OPRI, Y163X
BV	0.1	50	HEPES	8	NaI	300	0.002	30	20	Control CSF 2/4 wells positive > 60 hrs; negative for P102L, D178-FFI 6-OPRI, Y163X
Ha90	0.1	50	HEPES	8	NaI	300	0.002	30	20	Control CSF 2/4 wells positive ≥ 45 hrs; unstable
BV	0.1	55	PBS	7.4	NaCl	300	0.002	24	20	Control CSF 2/4 wells positive > 50 hrs; negative for P102L, D178-FFI, 6-OPRI, Y163X
BV	0.1	55	HEPES	8	NaI	300	0.002	24	20	Control CSF 4/4 wells low positive > 54 hrs; unstable
Ha90	0.1	55	HEPES	8	NaI	300	0.002	24	20	Control CSF 2/4 wells positive > 35 hrs; unstable
Hu	0.05	37	PIPES	7	NaI	500	0	50	5	Control CSF 3/4 wells positive > 45 hrs; unstable
Hu	0.05	37	PIPES	7	NaCl	500	0	50	5	Control CSF 1/4 wells positive > 70 hrs; P102L-Cog and 6-OPRI late positives, unable to replicate
Hu	0.1	42	PBS	7.4	NaCl	130	0.002	50	20	No spontaneous fibrillisation in Control CSF wells up to 85 hrs; negative for P102L, D178-FFI, 6-OPRI, Y163X
Hu	0.1	42	PBS	7.4	NaI	130	0.002	50	20	No spontaneous fibrillisation in Control CSF wells up to 85 hrs; negative for P102L, D178-FFI, 6-OPRI, Y163X
Hu*	0.05	37	PIPES	7	NaI	500	0	50	5	Control CSF 3/4 wells positive > 45 hrs; unstable
Hu*	0.05	37	PIPES	7	NaCl	500	0	50	10	No spontaneous fibrillisation in Control CSF wells up to 85 hrs; negative for P102L, D178-FFI, 6-OPRI, Y163X
Hu*	0.05	37	PIPES	7	NaCl	500	0.001	50	10	No spontaneous fibrillisation in Control CSF wells up to 85 hrs; negative for P102L, D178-FFI 6-OPRI, Y163X
Hu*	0.05	37	PIPES	7	NaI	500	0	50	8	Control CSF 3/4 wells positive ≥ 45 hrs; unstable
Hu P102L	0.05	37	PIPES	7	NaI	150	0	50	5	Control CSF 4/4 wells positive ≥ 45 hrs; unstable
Hu P102L	0.05	37	PIPES	7	NaCl	500	0	50	5	Control CSF 4/4 wells positive ≥ 45 hrs; unstable
Hu P102L	0.1	42	PBS	7.4	NaCl	130	0.002	50	20	No spontaneous fibrillisation in Control CSF wells up to 85 hrs; positive for subset of P102L-GSS
Hu P102L	0.1	42	PBS	7.4	NaI	130	0.002	50	20	No spontaneous fibrillisation in Control CSF wells up to 85 hrs; more sensitive than NaCl for P102L-GSS
BV90	0.1	55	PBS	7.4	NaCl	300	0.002	24	20	No spontaneous fibrillisation in Control CSF (pooled) wells up to 85 hrs; inhibitory factor?
BV90	0.1	55	HEPES	8	NaI	300	0.002	24	20	No spontaneous fibrillisation in Control CSF (pooled) wells up to 85 hrs; inhibitory factor?
BV90	0.1	55	PBS	7.4	NaCl	300	0.002	24	20	Control CSF 4/4 wells positive ≥ 43 hrs; unstable
BV90	0.1	55	HEPES	8	NaI	300	0.002	24	20	Control CSF 4/4 wells positive ≥ 20 hrs; unstable
BV	0.1	55	HEPES	8	NaI	300	0.002	24	15	Control CSF 4/4 wells positive ≥ 40 hrs; unstable

Hu* use 30s/30s shake/rest cycles (vs 60s/60s for all other experiments) in BMG microplate readers at 700 rpm; shaded rows represent best assay conditions available for testing symptomatic and IPD-AR CSF collection.
